# Clinical-grade cryopreserved doxorubicin-loaded platelets: role of cancer cells and platelet extracellular vesicles activation loop

**DOI:** 10.1186/s12929-020-00633-2

**Published:** 2020-03-23

**Authors:** Yu-Wen Wu, Cheng-Chain Huang, Chun Austin Changou, Long-Sheng Lu, Hadi Goubran, Thierry Burnouf

**Affiliations:** 1 Graduate Institute of Biomedical Materials and Tissue Engineering, College of Biomedical Engineering, Taipei Medical University, 250 Wu-Xing Street, Taipei, 11031 Taiwan; 2 Graduate Institute of Translational Medicine, College of Medicine, Taipei Medical University, Taipei, Taiwan; 3 The Ph.D. Program for Cancer Biology and Drug Discovery, Center for Translational Medicine, Taipei Medical University, Taipei, Taiwan; 4 International PhD Program in Biomedical Engineering, College of Biomedical Engineering, Taipei Medical University, Taipei, Taiwan; 5 Department of Radiation Oncology, Taipei Medical University Hospital, Taipei, Taiwan; 6 Translational Laboratory, Department of Medical Research, Taipei Medical University Hospital, Taipei, Taiwan; 7 International PhD Program in Cell Therapy and Regeneration Medicine, College of Medicine, Taipei Medical University, Taipei, Taiwan; 8 Saskatoon Cancer Centre and College of Medicine, University of Saskatchewan, Saskatchewan, Canada

**Keywords:** Cryopreserved platelet, Tissue factor, Cancer, Doxorubicin, Platelet extracellular vesicles, Drug delivery

## Abstract

**Background:**

Human platelets (PLT) and PLT-extracellular vesicles (PEV) released upon thrombin activation express receptors that interact with tumour cells and, thus, can serve as a delivery platform of anti-cancer agents. Drug-loaded nanoparticles coated with PLT membranes were demonstrated to have improved targeting efficiency to tumours, but remain impractical for clinical translation. PLT and PEV targeted drug delivery vehicles should facilitate clinical developments if clinical-grade procedures can be developed.

**Methods:**

PLT from therapeutic-grade PLT concentrate (PC; *N* > 50) were loaded with doxorubicin (DOX) and stored at − 80 °C (DOX-loaded PLT) with 6% dimethyl sulfoxide (cryopreserved DOX-loaded PLT). Surface markers and function of cryopreserved DOX-loaded PLT was confirmed by Western blot and thromboelastography, respectively. The morphology of fresh and cryopreserved naïve and DOX-loaded PLT was observed by scanning electron microscopy. The content of tissue factor-expressing cancer-derived extracellular vesicles (TF-EV) present in conditioned medium (CM) of breast cancer cells cultures was measured. The drug release by fresh and cryopreserved DOX-loaded PLT triggered by various pH and CM was determined by high performance liquid chromatography. The thrombin activated PEV was analyzed by nanoparticle tracking analysis. The cellular uptake of DOX from PLT was observed by deconvolution microscopy. The cytotoxicities of DOX-loaded PLT, cryopreserved DOX-loaded PLT, DOX and liposomal DOX on breast, lung and colon cancer cells were analyzed by CCK-8 assay.

**Results:**

15~36 × 10^6^ molecules of DOX could be loaded in each PLT within 3 to 9 days after collection. The characterization and bioreactivity of cryopreserved DOX-loaded PLT were preserved, as evidenced by (a) microscopic observations, (b) preservation of important PLT membrane markers CD41, CD61, protease activated receptor-1, (c) functional activity, (d) reactivity to TF-EV, and (e) efficient generation of PEV upon thrombin activation. The transfer of DOX from cryopreserved PLT to cancer cells was achieved within 90 min, and stimulated by TF-EV and low pH. The cryopreserved DOX-loaded PLT formulation was 7~23-times more toxic to three cancer cells than liposomal DOX.

**Conclusions:**

Cryopreserved DOX-loaded PLT can be prepared under clinically compliant conditions preserving the membrane functionality for anti-cancer therapy. These findings open perspectives for translational applications of PLT-based drug delivery systems.

## Background

Human platelets (PLT) are abundant anucleated blood cells that are instrumental in hemostasis and tissue repair mechanisms [1, 2]. However, there is an increasing recognition of the instrumental contribution of PLT to cancer progression. PLT enhances tumour growth by the release of growth factors, conceal circulating tumour cells from immune defense mechanisms, and prime metastasis [3–7]. In the tumour microenvironment, a detrimental PLT-cancer cell activation loop is ignited, orchestrated by several functional PLT membrane receptors capable of targeting and interacting with cancer cell surface markers [8, 9]. The bioreactivity of PLT membranes to cancer cells culminate into PLT activation and “tumour cell-induced PLT aggregation” (TCIPA) [10, 11], which is triggered by tumour’s tissue factor (TF). Further, cancer cell-derived extracellular vesicles expressing tissue factor (TF-EV), released in the tumour microenvironment, could induce PLT activation [12]. Activation also leads to alteration of the PLT membrane structure, exposure of procoagulant phosphatidylserine, generation of PLT-derived extracellular vesicles (PEV), conversion of prothrombin into active thrombin, resulting in the hypercoagulable state typically observed in cancer patients [13–15]. Reciprocally, PLT activated in the tumour microenvironment release growth factors and chemokines, either as free molecules or as entities entrapped in PEV, which favor tumour growth, angiogenesis, and metastasis [16–18]. The “vicious” pathogenic attraction existing between tumour cells and PLT membranes has recently stimulated the engineering of targeted PLT membrane-inspired nanocarriers aiming at delivering anti-cancer agents, following a so-called “Trojan Horse” strategy. In this approach, the PLT membrane machinery serves as a camouflage against the phagocytic system and is used to design smart drug delivery systems (DDS) with enhanced targeting ability to tumour cells [19–24]. This PLT mimicry strategy was indeed found in animal models to lower the uptake of synthetic nanoparticles by macrophages and to avoid nanoparticle-induced complement activation, and was also demonstrated to improve the targeting of nanoformulations to tumours [20, 22, 25–27]. For instance, doxorubicin (DOX)-loaded liposomes (liposomal DOX) functionalized by a few PLT membrane receptors, such as glycoprotein IIb-IIIa (GPIIb-IIIa), P-selectin, and GPIa-IIa, demonstrated enhanced targeting and binding capacity for highly metastatic breast cancer cells in both in vitro and in vivo models [26]. Functionalization with whole PLT membranes also improved the ability of nanocarriers to target diseased tissues [27]. Drug-loaded nanoparticle/nanogel preparations coated with whole PLT membranes exerted potent toxicity against tumour cells and lowered the risk of metastasis in animal models [20, 28, 29]. Those studies are crucial proof-of-concept and learning tools in understanding the capacity of the bioreactive PLT membrane machinery to target cancer cells. However, isolating PLT membranes and achieving nanoparticle coatings are highly challenging at production scale, since designing clinically compliant isolation and purification procedures of PLT membranes is difficult [24, 30]. In addition, PLT membrane isolation procedures may induce protein denaturation that may affect the targeting capacity and trigger immunogenicity [31]. Furthermore, PLT membrane-coated nanocarriers do not have the full biochemical cross-talk ability inherently exhibited by PLT themselves and remain biologically inert to biochemical signals present in the tumour microenvironment. We believe that, to overcome these drawbacks, using PLT themselves as drug carriers is currently the most realistic and rational approach for clinical translation [30]. We herein developed a process to prepare a stable PLT formulation that can be manufactured from therapeutic-grade autologous or allogeneic PLT concentrate (PC). The isolated PLT were loaded with DOX suspended in a licensed PLT additive solution (PAS) [32] to form fresh DOX-loaded PLT, and stored frozen at − 80 °C using a 6% dimethyl sulfoxide (DMSO)-based formulation [33–37] (cryopreserved DOX-loaded PLT). Importantly, the capacity to store DOX-loaded PLT provides a logistically convenient and clinically compliant procedure that can benefit from the existing expertise in blood collection and transfusion medicine. We also demonstrate that the loading process of DOX and the freezing for long-term storage and ease of treatment does not affect (a) PLT function, in particular the membrane integrity or reactivity to tumour cell-derived TF and thrombin, (b) the capacity to generate PEV, and (c) the targeting and transfer of DOX to cancer cells. Our study unveils the unique and superior ability of cryopreserved intact PLT to serve as a “Trojan Horse” bioreactive delivery carrier of cancer drugs, through generation of potent PEV and enhanced release at low pH and in the presence of cancer cell-derived TF-EVs.

## Materials and methods

### Therapeutic-grade PC collection

The Institutional Review Board of Taipei Medical University approved this study (TMU-JIRB no.: 201502019). Over 50 clinical-grade PC, collected in 100% plasma, were obtained from the Taipei Blood Center (Taiwan Blood Services Foundation, Guandu, Taiwan). PC was obtained from volunteer healthy donors. Apheresis PC was prepared using the licensed Haemonetics MCS+ cell separator (Haemonetics Corp., Braintree, MA, USA) and whole blood-derived PC by the “platelet-rich-plasma” method. PC was anti-coagulated using a licensed citrate phosphate dextrose solution. All donations were tested and found to be non-reactive against mandatory viral markers, following local and international regulations. The PC was delivered within 3 days after collection to our laboratory where they were kept at 22 ± 2 °C on a platelet agitator and processed on the same day or within 9 days of collection. PC was centrifuged at 1500 xg for 10 min to separate PLT and plasma. Plasma was also collected after additional centrifugation at 3000 xg for 10 min twice to remove residual PLT. PLT and plasma were analyzed by ABC Vet (ABX Diagnostics, Montpellier, France) to determine the blood cell count.

### Preparation of fresh and cryopreserved DOX-loaded PLT

PC was centrifuged at 1500 xg for 10 min at 23 ± 1 °C to pelletize PLT. PLT was then gently resuspended in a solution of 100 μM DOX hydrochloride (LC Laboratories, Woburn, MA, USA) solubilized in PAS. The preparation was incubated gently on a suspension mixer (SM-3000; Yihder, Taipei, Taiwan) at 30 ± 5 rpm for 1 h at 37 ± 1 °C. Excess DOX was removed by centrifugation at 1500 xg for 10 min at 23 ± 1 °C. The pellets were gently resuspended in PAS to generate fresh DOX-loaded PLT or resuspended with 6% DMSO (Sigma-Aldrich, ST. Louis, MO, USA) to prepare cryopreserved DOX-loaded PLT formation.

### Characterization of fresh and cryopreserved DOX-loaded PLT

The concentration of DOX loaded in PLT isolated from PC stored from 3 to 9 days after collection was quantified. Preparation of DOX-loaded PLT is described above. 1 mL of DOX-loaded PLT pellets (ca. 1 × 10^9^ PLT) were lysed with 5% sodium dodecylsulfate (SDS; Sigma-Aldrich) and the concentration of loaded DOX in the PLT was determined by fluorospectrometer (470/585 nm).

### Deconvolution fluorescence microscopy of DOX-loaded PLT

Loading of DOX in PLT was visualized by immunofluorescence staining and observed by deconvolution microscopy. Degreased coverslips were coated with 200 μL of 10 mg/ml fibrinogen (Sigma-Aldrich) in saline at 22 ± 2 °C for 30 min [38]. 2 × 10^8^ DOX-loaded PLT were loaded onto the coverslip at 22 ± 2 °C for 30 min and fixed with 2 mL 4% paraformaldehyde for 10 min followed by blocking with 200 μL 2% bovine serum albumin (BSA; Sigma-Aldrich) in phosphate-buffered saline (PBS; Hyclone, Logan, UT, USA). CD41a PLT surface markers were stained with 200 μL of mouse anti-human CD41a-APC (BD Bioscience, San Jose, CA, USA) at a 1:20 dilution in 2% BSA for 1 h. The coverslips were mounted onto microscopic slides with 10 μL Everbrite mounting medium with DAPI (Biotium, Hayward, CA, USA) and visualized by DeltaVision Personal Deconvolution Microscopy (GE Healthcare, Marlborough, MA, USA). Three digital (3D) images were generated by Volocity 6.3 software (PerkinElmer, Waltham, MA, USA).

### PLT morphology observed by scanning electron microscopy (SEM)

To prove that freezing and drug loading did not affect the activation capacity, the morphology of PLT was observed by SEM (SU3500; Hitachi, Tokyo, Japan), using coverslips were coated with 10 mg/mL of fibrinogen (Sigma-Aldrich). Fresh and cryopreserved PLT and DOX-loaded PLT were loaded on the coverslips, and compared visually to DOX-loaded PLT loaded on the coverslip and activated with 0.1 U/mL thrombin (Sigma-Aldrich; 37 ± 1 °C, 30 min). Samples were fixed with 2% paraformaldehyde (Fluka, Buchs, Switzerland) and 2.5% glutaraldehyde (Sigma-Aldrich) in 0.2 M cacodylate (Sigma-Aldrich) for 30 min, dehydrated using 70~100% ethanol gradient solutions, and gold-coated.

### Western blot analysis of PLT membrane receptors

Protein concentrations of PLT samples were determined by a Bradford protein assay kit (Thermo-Fisher Scientific, Waltham, MA, USA). 10 μg of protein of each samples were mixed with 4x sample buffer and heated at 70 °C for 10 min before separation by SDS-polyacrylamide gel electrophoresis using a 4~12% gradient gel. Proteins were transferred to polyvinylidene difluoride (Pall Corp., Port Washington, NY, USA) membranes, and blocked using 5% skim milk. Membranes were incubated with antibodies against PLT glycoprotein IIb (CD41; Abcam, Cambridge, MA, USA), PLT glycoprotein IIIa (CD61; Abcam), protease activated receptor-1 (PAR-1, R&D Systems, Minneapolis, MN, USA), and glyceraldehyde 3-phosphate dehydrogenase (GAPDH) as the loading control (Novus Biologicals, Littleton, CO, USA). Horseradish peroxidase-conjugated antibodies (Jackson ImmunoResearch Laboratories, West Grove, PA, USA) were used as a secondary antibody. The detailed information of the antibodies used is in Table S2. Detection was done by enhanced chemiluminescence (ECL) (GE Healthcare). Bands were analyzed on a BioSpectrum 810 Imaging System (Analytik Jena, Jena, Germany).

### PLT function test

The PLT function was assessed by thromboelastometry (TEG 5000 Thrombelastograph Hemostasis Analyzer System, Haemonetics). Samples were diluted with plasma to a content of 4 x 10^8^ PLT/mL, and mixed with 40 μL kaolin, after which 330 μL was placed in a cup with 30 μL of 0.2 M calcium chloride (CaCl_2_; Sigma-Aldrich) for the thromboelastography analysis [39]. Platelet-poor plasma was used as a negative control. The following tracing parameters were evaluated: the reaction time (R) to initial fibrin generation and the maximum amplitude (MA) to assess the maximum strength of clot formation, as described by the supplier.

### In vitro drug release profile

#### Impact of pH

The in vitro release of DOX from loaded PLT was determined in PAS at pH 7.4 (control) and in PBS at pH 7.4, 6.5, or 5.5. DOX-loaded PLT (4 × 10^8^ PLT) were suspended in 0.4 mL of a test solution, transferred into 1.5 mL Eppendorf tubes, and placed on a shaker (TS-500, Yihder Co.) at 50 rpm and 22 ± 2 °C. Samples were taken at the start and after 6, 12, 24, 48, and 72 h of incubation. The suspension was centrifuged at the given time points in order to determine the residual amount of DOX still present in PLT. The pellet was lysed with 5% SDS and diluted 5-fold by 40% acetonitrile. The concentration of DOX was quantified by high performance liquid chromatography (HPLC; Hitachi) equipped with an Symmetry C18 column (4.6 × 150 mm, 3.5 μm analytical column; Waters, Milford, MA, USA) maintained at 22 ± 2 °C. Chromatography was run at 1 mL/min in 40% acetonitrile at pH 2.9 (citric acid monohydrate, Sigma-Aldrich) using a 470/585-nm fluorescence detector [40]. The calibration standards of DOX was prepared in 40% acetonitrile at concentration of 50, 100, 200, 500, 2000, 4000, 6000 ng/mL. The release profile of DOX from fresh or cryopreserved PLT was calculated according to the formula:

$$ \mathrm{Cumulative}\ \mathrm{DOX}\ \mathrm{release}\ \left(\%\right)=\left[1-\left(\frac{\mathrm{Amount}\ \mathrm{of}\ \mathrm{DOX}\ \mathrm{in}\ \mathrm{PLT}\ \mathrm{at}\ \mathrm{time}\ \mathrm{point}}{\mathrm{Initial}\ \mathrm{amount}\ \mathrm{of}\ \mathrm{DOX}\ \mathrm{in}\ \mathrm{PLT}}\right)\right]\mathrm{x}\ 100\% $$

### Impact of conditioned medium cultured with breast cancer cells

In vitro release of DOX was measured when DOX-loaded PLT was incubated in the supernatant of MDA-MB-231 cell conditioned medium (CM) to mimic components of the tumour microenvironment. The release kinetics was compared to that determined when incubation was done in control medium (Dulbecco’s modified Eagle medium; DMEM). Loaded PLT was suspended in culture medium and mixed on a shaker at 50 rpm and 37 °C. At each pre-determined time points (0, 6, 12, 24, 48 and 72 h), the mixture was centrifuged (1500 xg for 10 min at 23 ± 1 °C), and the supernatant was separated from the pellet. The concentration of DOX was determined by HPLC (Hitachi) and the formula of kinetic release of DOX was calculated as described above.

### Cell experiments

MCF-7 (BCRC-60436; Bioresource Collection and Research Center, Hsin-Chu, Taiwan) and MDA-MB-231 (BCRC-60425) human breast cancer cells were grown in DMEM (Hyclone) supplemented with 10% fetal bovine serum (FBS, Hyclone). The A549 (BCRC-960402) human lung cancer cell line was maintained in F12K medium (Gibco, Grand Island, NY, USA) supplemented with 10% FBS. The HCT 116 (ATCC-CCL-227) human colon cancer cell line was preserved in McCoy’s 5a modified medium (Gibco) supplemented with 10% FBS. All cells were incubated at 37 °C in a 5% CO_2_ atmosphere.

### Cellular uptake of DOX-loaded PLT

Microscopic observations and time-lapse of transfer to cancer cell nuclei was performed as follows. 10^5^ MCF-7 cells/well were seeded on coverslips in six-well plates and maintained at 37 °C overnight. Approximately 2 × 10^8^ DOX-loaded PLT were added, and the mixture was maintained at 37 °C for 3 h, then fixed using 4% paraformaldehyde (Fluka) for 10 min at 22 ± 2 °C, and washed with PBS (Hyclone) three times. Fixed cells were permeabilized using 0.1% Triton X-100 (Merck Millipore, Darmstadt, Germany) for 4 min at 22 ± 2 °C and blocked with 1% BSA at 24 ± 2 °C for 30 min. Cells were then incubated with phalloidin Alexa-Fluor 647 (Life Technologies, Eugene, OR, USA) at a 1:40 dilution in 1% BSA for 1 h to stain F-actin. Nuclei were stained by Mounting Medium with DAPI (Biotium). Cells were visualized by three-dimensional (3D) images by deconvolution fluorescence microscopy and analyzed by Volocity software (PerkinElmer). Further, DOX transfer from PLT to cells was observed for 90 min in real time (Figure S1). Briefly, 10^5^ MCF-7 cells were seeded on coverslips in glass-bottom dishes and maintained at 37 ± 1 °C overnight. 2 × 10^8^ DOX-loaded PLT were added to the dish, and then images were taken by deconvolution fluorescence microscopy over time, and a movie was generated with Volocity software (PerkinElmer).

### TF activity of conditioned medium cultured with breast cancer cells

MDA-MB-231 cells (5 × 10^6^) were seeded in a T-75 flask and grown for 24 h in DMEM (Hyclone) supplemented with 0.2 μm-filtered 10% FBS (Hyclone). The medium was then replaced with 10 mL of complete culture medium, and cells were cultured for an additional 48 h. Cell culture supernatants were centrifuged at 300 xg to remove cells, then 3000 xg for 10 min at 23 ± 1 °C to remove cell debris. Supernatants of cancer cells (CM) and culture medium (DMEM) control were collected and frozen at − 80 °C until the in vitro drug release test and MP-TF activity assay. The presence of TF-EV in the medium was determined by a functional Zymuphen MP-TF capture assay (Hyphen BioMed, Neuville sur Oise, France) that measures the procoagulant activity of EV expressing TF. The endpoint absorbance was measured at 405 nm using a microplate reader (TECAN Trading AG, Mannedorf, Switzerland).

### TF activity and thrombin generation assay in the conditioned medium of MDA-MB-231 cells incubated with cryopreserved PLT

1 × 10^6^ MDA-MB-231 cells were pelletized by centrifugation at 200 xg at 23 ± 1 °C. Cell pellets were resuspended with 1 mL of plasma or with 3 × 10^9^ cryopreserved PLT in plasma. In addition, plasma or 3 × 10^9^ cryopreserved PLT in plasma were used as controls. All samples were incubated at 37 °C for 1 h with stirring at 70 rpm on a shaker (Yihder). Intact cells were removed by centrifugation at 300 xg for 10 min followed by 3000 xg for 10 min to remove cell debris and PLT. The supernatant of each sample was collected to assess TF activity associated with EV (“MP-TF activity” assay) leading to thrombin generation. The generation of thrombin was measured by a fluorogenic substrate induced by TF and negatively charged phospholipid-containing EV. For the thrombin generation assay (Technoclone, Vienna, Austria), samples were incubated with a low concentration of phospholipid micelles containing recombinant human TF following manufacturer’s instructions. 40 μL of sample was added into 96 well-plate followed by addition of 50 μL of a mixture of the RCL reagent and the substrate. The fluorescence (360 nm/460 nm) change was measured by spectrophotometer (Thermo-Fisher Scientific).

### Isolation of DOX-loaded PEV

200 mL of PC was centrifuged at 3000 xg for 10 min at 22 ± 1 °C to obtain PLT pellet. The PLT pellet was suspended with thrombin in 200 mL Tyrode’s buffer (final concentration, 0.1 U/ mL) to trigger activation, and incubated at 37 °C for 30 min. The activation was stopped by 0.5 M EDTA in Tyrode’s buffer (final concentration, 20 mM). The solution was then centrifuged at 3000 xg for 10 min at 24 ± 3 °C to remove PLTs. The supernatant was centrifuged at 20000 xg for 90 min at 18 °C, as described in a previous study [41]. The PEV pellet was re-suspended with 2 mL PAS and stored at − 80 °C until use. 100 μL PEV were thawed at 37 °C then incubated with 900 μL of 100 μM DOX in PAS at 37 °C for 1 h. The solution was centrifuged at 20000 xg for 90 min at 18 °C. DOX-loaded PEV (PEV-DOX) pellet was re-suspended with 1 mL of PAS for further tests.

### Characterization of DOX-loaded PEV

To observe the delivery of DOX to cells by DOX-loaded PEV, 1x10^5^/ well MCF-7 and MDA-MB-231 were seeded on coverslips in 6-well plates at 37°C overnight. Approximate 200 μL of 1x10^8^ DOX-loaded PEV were deposited on coverslips and maintained at 37°C for 30 min. The samples were then fixed with 1 mL of 4% PFA for 10 minutes at 24 ± 3°C and washed with 1 mL 1x PBS three times. The samples were blocked with 200 μL 1% BSA at RT for 30 min prior to incubation with 200 μL CD62P at a concentration of 1:20 in 1% BSA for 1 h to stain PEV. The coverslips were mounted onto slides with 10 μL Mounting Medium with DAPI to stain nuclei and visualized 3D images by deconvolution fluorescence microscopy. 1 mL of isolated PEV and PEV-DOX in PAS were measured by nanoparticle tracking analysis (NTA; Nanosight NS300 system; Malvern, UK) with a 488-nm laser and CCD camera (sCMOS). Samples were processed as described above. The syringe pump speed was set to 50. The data were analyzed three times during 60 s at 24 ± 1 °C, and the videos were recorded. Data were analyzed with NTA software 3.1 as provided by the supplier (Malvern).

### In vitro cell proliferation and cytotoxicity assay

100 μL of 5 × 10^4^ MCF-7 and MDA-MB-231 cells/mL were seeded in 96-well plates overnight and subsequently treated with DOX-loaded PLT, DOX and liposomal DOX (TTY Biopharm, Taipei, Taiwan). After 48 h of incubation, cell proliferation was measured with a 5-bromo-2′-deoxyuridine (BrdU) cell proliferation enzyme-linked immunosorbent assay (ELISA; Roche Diagnostics, Mannheim, Germany). In addition, the in vitro cytotoxicity was assessed by a Cell Counting Kit-8 (CCK-8; Sigma-Aldrich) assay. 100 μL of 5 × 10^4^ A549, HCT 116 and MDA-MB-231 cells/mL were seeded in 96-well plates at 37 ± 1 °C and treated with fresh and cryopreserved DOX-loaded PLT, free DOX, or liposomal DOX for 48 h. The CCK-8 solution was added to each well and incubated for 3 h at 37 ± 1 °C. Both assays of plates were read at 450 and 690 nm as reference wavelengths in a spectrometer. They were calculated as a percent of the control using the following formula: Cell proliferation (%) = (optical density (OD) of the experimental group / OD of the control group) × 100.

### Statistical analysis

All tests were done in at least three independent experiments and using different batches of fresh and cryopreserved DOX-loaded PLT to ensure reproducibility. Results are expressed as the mean ± standard deviation (SD). The statistical analyses were performed using GraphPad Prism 6 software with a one-way ANOVA, two-way ANOVA or a paired t-test. A *p* value < 0.05 was considered the be significant (* *p* < 0.05, ** *p* < 0.01, *** *p* < 0.001, **** *p* < 0.0001, ns = non-significant).

## Results

### DOX loading procedure and characterization

**Fig. 1:**
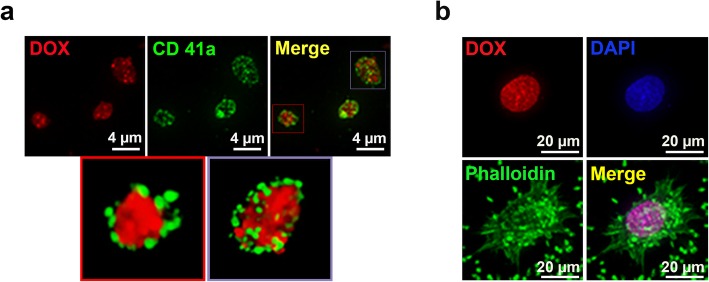
Characterization of fresh DOX-loaded PLT. (**a**) Deconvolution fluorescence images revealed the presence of DOX (red) within fresh PLT stained with PLT membrane marker GPIIb (CD41a). (**b**) Deconvolution fluorescence images of MCF-7 breast cancer cells incubated with DOX-loaded PLT; nuclei were stained with DAPI (blue) and actin of MCF-7 and PLT with phalloidin (green)

Over 50 clinical-grade human PC were produced by licensed blood collection procedures. The PC contained 1.24 ± 0.27 × 10^9^ PLT/mL, 1.18 × 10^6^ ± 1.29 × 10^6^/mL white blood cells, and 8.3 ± 8.5 × 10^7^/mL red blood cells. Three to nine days after collection, PC was centrifuged at 1500 xg for 10 min to isolate the PLT and remove plasma. The isolated PLT was suspended in a PAS. To prepare DOX-loaded PLT, PLT was incubated for 1 h at 37 ± 1 °C in 100 μM of DOX solubilized in PAS. DOX loading within PLT by this passive transfer was demonstrated by fluorescence microscopy and 3D deconvolution microscopic images (Fig. 1a). Storing the PC for up to 9 days after collection at 22 ± 2 °C increased the quantity of DOX loaded from 30.71 μM at day 3 to 49.78 μM at day 9, corresponding to 15 to 36 × 10^6^ molecules of DOX per PLT (Table S1). The quantity of DOX loaded increased from day 3 to day 6 after PC collection, then reached a plateau.

Evidence of the capacity of the freshly loaded PLT to transfer DOX to cancer cells nuclei was first checked by time-lapse microscopy. DOX transfer was concomitant with binding of PLT to cancer cell membranes (Fig. 1b) and was completed as quickly as 90 min of incubation (Fig. S1), strongly suggesting that MCF-7 cells released factors stimulating PLT activation and DOX release. The BrdU assay to compare the in vitro cytotoxicity of DOX-loaded PLT, DOX, and liposomal DOX found mean respective 50% inhibitory concentration (IC50) values (expressed in DOX equivalents) at 48 h of 0.14 μM for free DOX and DOX-loaded PLT, and 2.31 μM for liposomal DOX with MCF-7, and 0.25, 0.24, and 2.25 μM, respectively, with MDA-MB-231 cells (Fig. S2). Therefore, these human PLT could readily and reproducibly be loaded with DOX and the toxicity of the resulting DOX-loaded PLT formulation was similar to that of free DOX and even over 10 times stronger than that of liposomal DOX*.*

### Freezing of DOX-loaded PLT

**Fig. 2:**
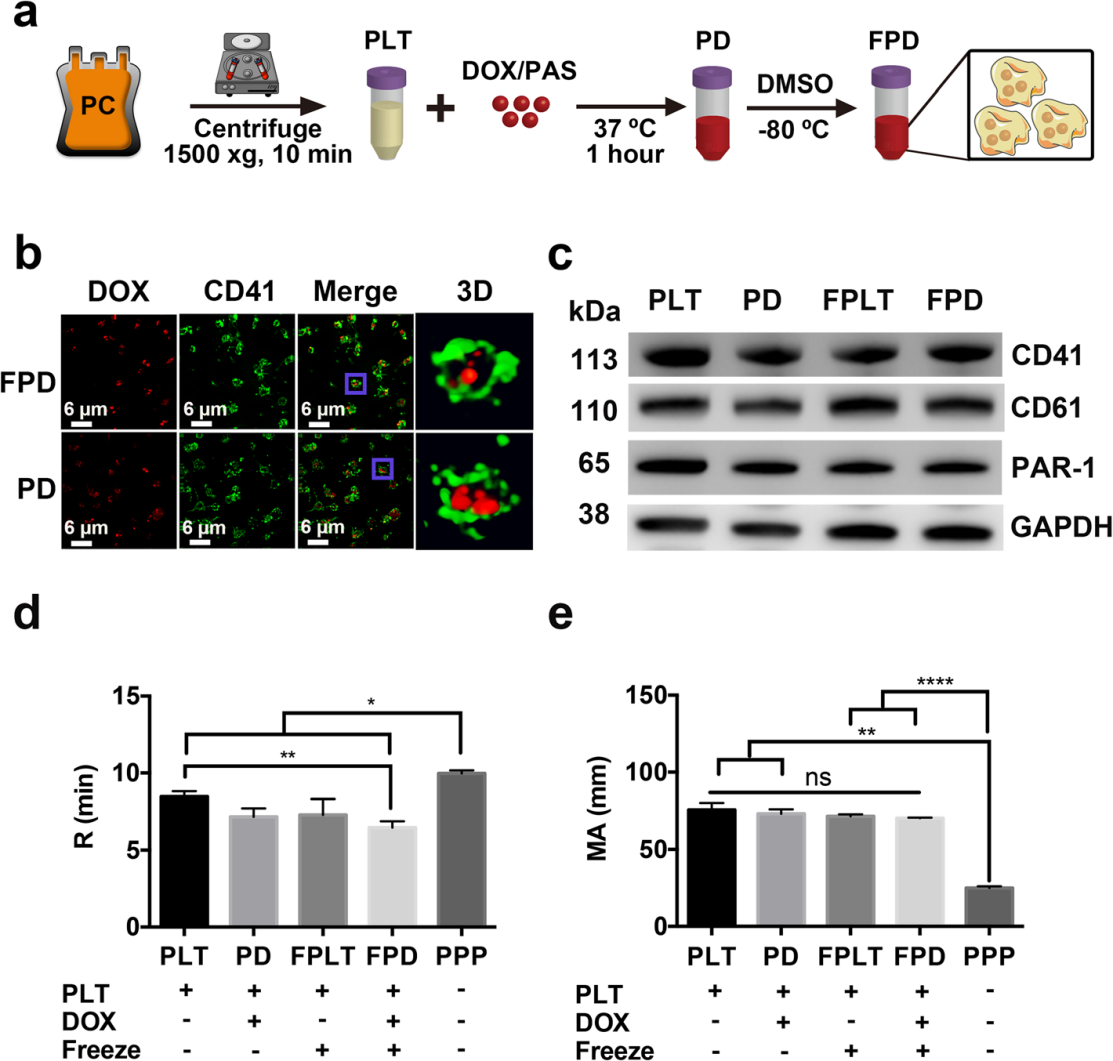
Characterization of fresh and cryopreserved DOX-loaded PLT. (**a**) Scheme of preparation of PD and FPD. (**b**) Fluorescent images of FPD compared to PD. (DOX: red, PLT membrane marker: green) (**c**) Western blot analysis showing similar expression of membrane surface markers: CD41, CD61, and PAR-1 in PLT, DOX-loaded PLT, cryopreserved PLT and cryopreserved DOX-loaded PLT; GAPDH was used as a control. The PLT functional assay of R time (**d**) and MA values (**e**) of PLT, DOX-loaded PLT, cryopreserved PLT, cryopreserved DOX-loaded PLT, and platelet-poor plasma (PPP) determined by PLT functional thromboelastography assay. * *p* < 0.05, ** *p* < 0.01, ****p < 0.0001, ns = non-significant. *Abbreviations: PC: platelet concentrate, PLT: platelet, DOX: doxorubicin, PAS: PLT additive solution, PD: DOX-loaded PLT, FPD: cryopreserved DOX-loaded PLT*

To facilitate clinical translation and administration schedule to patients, it is important that DOX-loaded PLT can be stored over the duration of treatment. Therefore, we evaluated the possibility to freeze and store DOX-loaded PLT at − 80 °C in the presence of 6% DMSO as it is a cryoprotectant commonly used to store stem cells in similar concentrations for stem cell transplantation. Cryopreserved DOX-loaded PLT was prepared as Fig. 2a. Deconvolution microscopy demonstrated that DOX could still be visualized within PLT after the freeze and thaw cycle (Fig. 2b), and Western blot identified that the expression of functionally important PLT membrane receptors (CD41, CD61, and PAR-1) was also preserved and similar to that in fresh DOX-loaded PLT (Fig. 2c). The morphologies of fresh DOX-loaded PLT and cryopreserved DOX-loaded PLT in SEM were similar to those of PLT and cryopreserved PLT with no evidence of formation of pseudopods, a visual marker of PLT activation [42], as observed in the thrombin-treated samples (Fig. S3). The thromboelastography functional assay was then used to evaluate the hemostatic activity and contribution of PLT to the strength of fibrin clots. The reaction time (R time, Fig. 2d) for DOX-loaded PLT was 7.17 ± 0.35 min and 6.47 ± 0.42 min for cryopreserved DOX-loaded PLT, both significantly (*p* < 0.01) shorter than that of 8.47 ± 0.35 min for PLT, but not significantly different (*p* > 0.05) from one another. More importantly, maximum amplitude (MA) values of DOX-loaded PLT or cryopreserved DOX-loaded PLT, a parameter directly linked to the functional contribution of PLT to fibrin clots strength, did not significantly differ from each other (p > 0.05) or from that of normal PLT (Fig. 2e). As expected, MA values of cryopreserved DOX-loaded PLT were significantly higher (*p* < 0.0001) than that of PLT-poor plasma (negative control). Thus, loading of DOX in PLT and subsequent freezing and thawing did not significantly affect the hemostatic function of PLT associated with the activity of the GPIIb/IIIa receptor, which is a key integrin in the PLT-cancer interaction loop.

### Drug release of fresh and cryopreserved DOX-loaded PLT in various pH and cancer conditioned medium

**Fig. 3:**
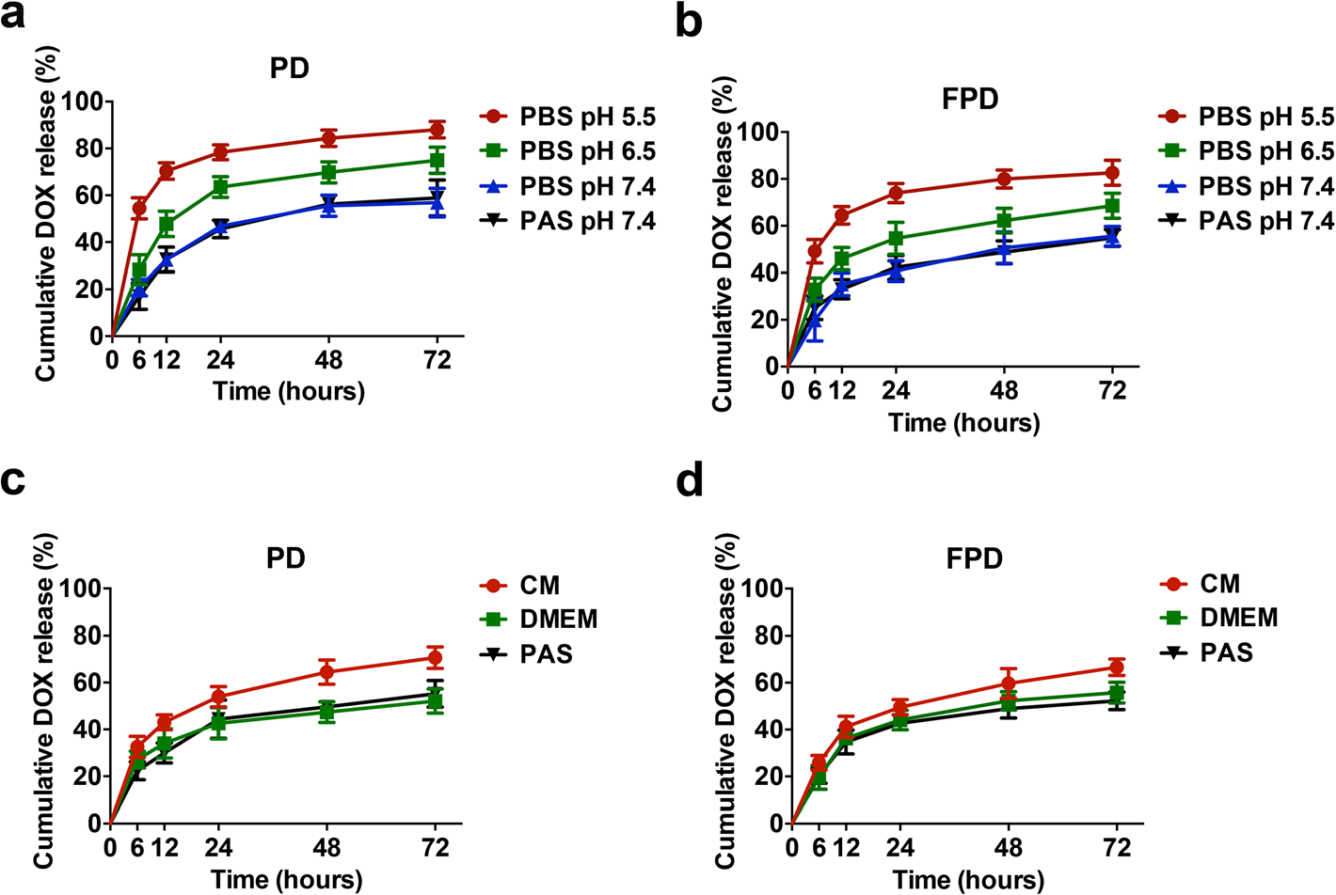
Drug release profiles of fresh and cryopreserved DOX-loaded PLT. Comparison of the kinetics of the release of DOX from DOX-loaded PLT (**a**) and cryopreserved DOX-loaded PLT (**b**) in PBS at pH 5.5, 6.5, and 7.4 and PAS at pH 7.4. Comparison of the kinetics of the release of DOX from DOX-loaded PLT (**c**) and cryopreserved DOX-loaded PLT (**d**) in the presence of CM, DMEM and PAS. *Abbreviations: DOX: doxorubicin, PLT: platelet, PD: fresh DOX-loaded PLT, FPD: cryopreserved DOX-loaded PLT, CM: conditioned medium cultured with cancer cells, DMEM: cell culture control medium, PBS: phosphate-buffered saline, PAS: PLT additive solution*

To assess the maintenance of the bioreactivity of the membrane of the loaded PLT, we determined how pH influenced the release kinetics of DOX in vitro*,* as lower pH is a characteristic of the tumour microenvironment. DOX-loaded PLT were transferred into Eppendorf and residual DOX still present in PLT was quantified over-time (we did not use a dialysis membrane as it could cause PLT adhesion, activation and aggregation potentially resulting in artefactual drug release). The cumulative amount of DOX released by DOX-loaded PLT or cryopreserved DOX-loaded PLT in PAS or phosphate-buffered saline (PBS) gradually increased over 72 h. It reached approximately 56% at pH 7.4 in PAS and PBS, implying that these two formulations did not impact, at least at pH 7.4, the kinetics of DOX release, nor did the additional freeze-thaw process used to prepare cryopreserved DOX-loaded PLT. Approximately 21% of the initial DOX content was still present within PLT after 6 days in PAS at pH 7.4 (Fig. S4). In contrast, lower pH significant fastens (*p* < 0.0001) the release of DOX. The mean total percentages of DOX released from DOX-loaded PLT in PBS over 72 h reached approximately 56.91% at pH 7.4, 74.93% at pH 6.5, and 88.03% at pH 5.5 (Fig. 3a). The percentages of DOX released from cryopreserved DOX-loaded PLT in PBS reached 82.60% at pH 5.5, 68.57% at pH 6.5, and 55.54% at pH 7.4 (Fig. 3b). Thus, the release of DOX by both cryopreserved DOX-loaded PLT and DOX-loaded PLT was pH-dependent and enhanced by low pH.

We then performed cancer cell cultures and collected the conditioned medium and checked for the presence of cancer cell-derived extracellular vesicles expressing tissue factor (TF-EV), as TF is known to be a key factor of PLT activation in cancer patients [43, 44]. The supernatant of the conditioned medium of MDA-MB-231 cancer cells (MDA-MB-231-EV) had a content of TF-EV (468.90 ± 54.15 pg/mL) significantly higher (*p* < 0.0001) than that of its control medium (DMEM; not detectable) (Fig. S5). DOX release from DOX-loaded PLT exposed to MDA-MB-231-EV for 72 h was significantly (p < 0.0001) higher (70.61%) than when they were exposed to DMEM (52.16%) or PAS (55.22%) (Fig. 3a). The DOX release profile from cryopreserved DOX-loaded PLT was also significantly (*p* < 0.001) higher in MDA-MB-231-EV (66.67%) than in DMEM (55.83%) or PAS (52.28%) (Fig. 3d). Thus, cancer cell released TF-EV and stimulated the release of DOX from DOX-loaded PLT and cryopreserved DOX-loaded PLT. Approximately 33% of DOX was present within PLT after 72 h of exposure to MDA-MB-231-EV (Fig. 3d), indicating that PLT can serve as a long-term DOX delivery system. Thus we observed an enhanced release of DOX from DOX-loaded PLT by exposure to low pH and TF-EV, used to mimic conditions present in the tumour environment.

### MDA-MB-231 cell-derived TF-EV induce thrombin generation resulting in PLT activation and PEV release

**Fig. 4:**
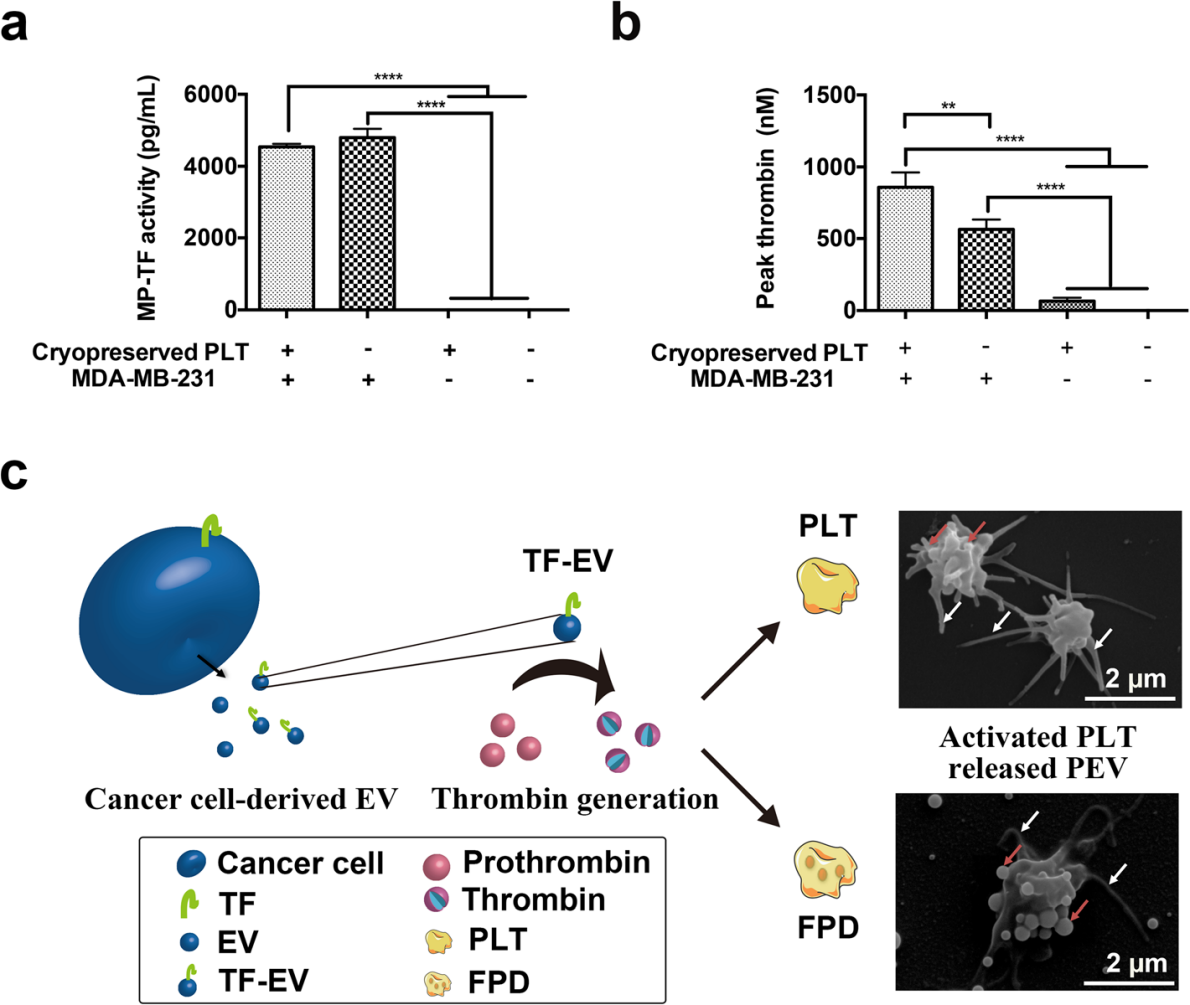
MDA-MB-231 cells trigger PLT activation and thrombin generation. (**a**) MP-TF activity and (**b**) thrombin generation induced by cryopreserved PLT with MDA-MB-231 cells, MDA-MB-231 cells, cryopreserved PLT and PPP, as measured by MP-TF activity assay and thrombin generation assay, respectively. (**c**) Schematic representation of the mechanisms through thrombin induced activation of cryopreserved DOX-loaded PLT compared to PLT to form peudopodia (white arrow) and release of PEV (red arrow). (** *p* < 0.01, **** *p* < 0.0001. *Abbreviations: PC: platelet concentrate, PLT: platelet, DOX: doxorubicin, FPD: cryopreserved DOX-loaded PLT, PEV: PLT-derived extracellular vesicle. PPP: PLT poor plasma*

MDA-MB-231 cell-derived TF-EV has been shown to activate PLT through thrombin generation in plasma [12]. Therefore, here, MDA-MB-231 cells were incubated with cryopreserved PLT. We assessed (a) their capacity to generate TF-EV using a specific TF activity assay and (b) induce thrombin generation by a thrombin generation assay. The mean TF activity of MDA-MB-231 cells incubated with cryopreserved PLT for 1 h in plasma reached ca. 4800 ± 245 pg/mL, and MDA-MB-231 cells in plasma for 1 h, was still ca. 4541 ± 87 pg/mL (Fig. 4a). Both values were significantly higher (*p* < 0.0001) than that of cryopreserved PLT and plasma controls (1.35 ± 0.07 and 1.18 ± 0.2 pg/mL, respectively). The MDA-MB-231-EV induced thrombin formation (Fig. 4b). The mean peak of thrombin generation was ca. 856 ± 103 nM, significantly higher (*p* < 0.01) than that by MDA-MB-231-EV not exposed to cryopreserved PLT (ca. 565 ± 68 nM), and significantly higher (p < 0.0001) than that by cryopreserved PLT alone (ca. 62 ± 23 nM) and plasma (undetectable). Thus, cancer cells incubated with PLT in plasma induced TF-EV expression and thrombin generation resulting in PLT activation.

We were then interested in elucidating the contribution of PEV in the transfer of DOX to cancer cells. Both PLT and cryopreserved DOX-loaded PLT exposed to thrombin could be activated, as evidenced in SEM by the formation of pseudopods (Fig. 4c). Thrombin-activated cryopreserved DOX-loaded PLT released PEV of approximately 100~250 nm as assessed by scanning electron microscopy (Fig. 4c, bottom right figure). This indicated that the surface membrane receptors on cryopreserved DOX-loaded PLT remained functional, leading to the capacity to induce PLT activation in response to thrombin agonist stimulation. Thus, to sum-up, MDA-MB-231 cells incubated with cryopreserved PLT induced the release of TF-EV and thrombin generation resulting in PLT activation and subsequent release of PEV.

### Isolation of DOX-loaded PEV and DOX delivery to cancer cells

**Fig. 5:**
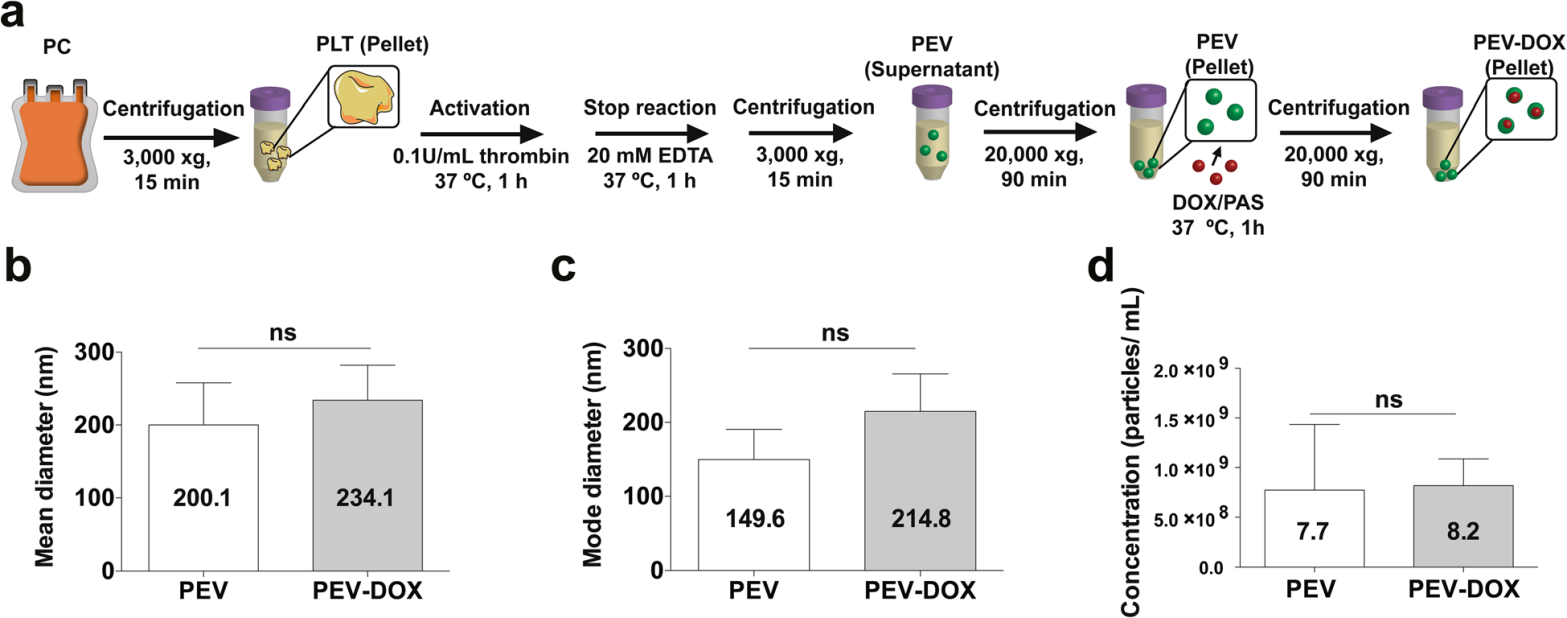
Size and concentration of isolated DOX-loaded PEV analyzed by NTA. (**a**) Scheme of isolation of PEV-DOX compared to PEV. (**b**) The mean diameter, (**c**) mode diameter and (**d**) concentration of PEV-DOX compared to PEV measured by NTA. ns = non-significance. *Abbreviations: PLT: platelet, PEV: PLT extracellular vesicle, DOX: doxorubicin, PEV-DOX: DOX-loaded PEV, NTA: nanoparticle tracking analysis*

**Fig. 6:**
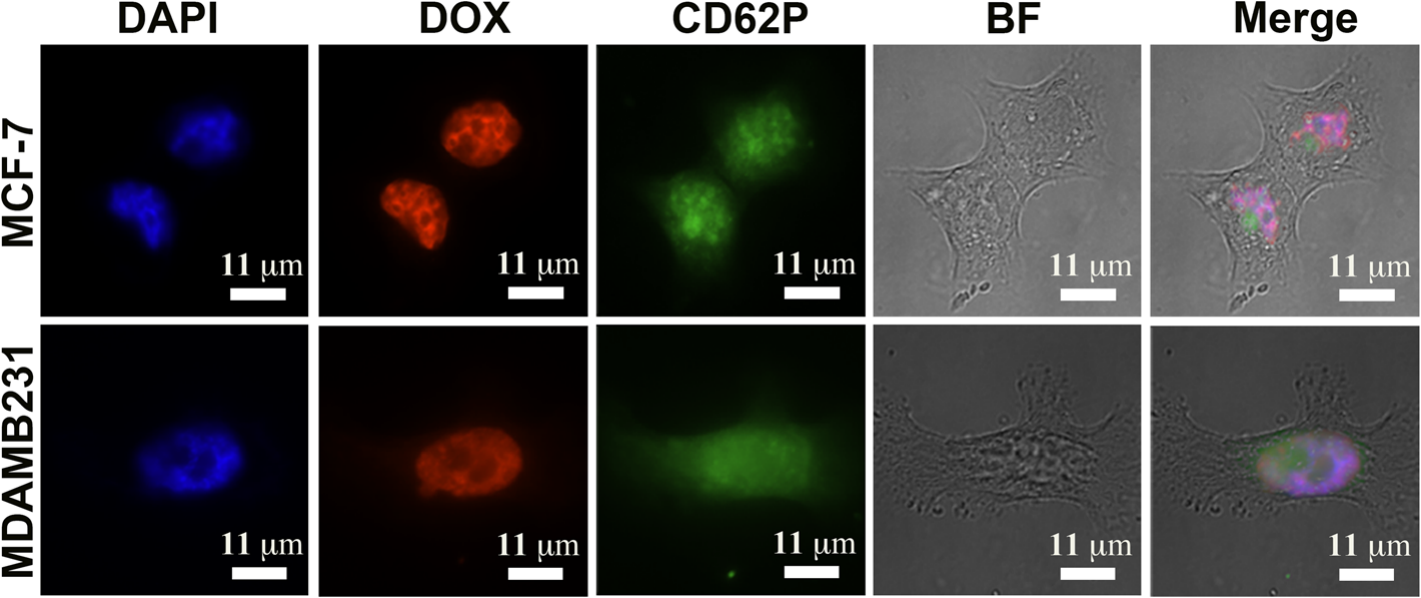
Deconvolution fluorescence image of breast cancer cell lines MCF-7 and MDA-MB-231 incubated with PEV-DOX. DOX was in red. Nuclei were stained with DAPI in blue, and PEV by CD62P in green. Cell morphology was visualized under bright field. The merged images showed the localization of DOX, cell nuclei and PEV. *Abbreviations: DOX: doxorubicin, PLT: platelet, BF: bright field, PEV- DOX: DOX-loaded PLT extracellular vesicle*

It has been shown that PEV can infiltrate solid tumours and transfer their content to cancer cells [45]. We therefore elucidated further the functional activity of DOX-loaded PEV generated by thrombin activation of unloaded PLT, and examined their capacity to deliver DOX to cancer cells. PEV-DOX were compared to unmodified PEV (Fig. 5a). The NTA analysis showed that mean size of PEV-DOX (234.1 ± 48.01 nm) was slightly larger than that of PEV (200.1 ± 57.71 nm) and that DOX entrapment did not influence the physical properties of the PEV (Fig. 5b, c and d). The size distribution profiles and particle images of PEV and PEV-DOX are shown in Fig. S6. When incubated with MCF-7 and MDA-MB-231, PEV-DOX transferred DOX to cancer cells nuclei within 30 min (Fig. 6). Therefore, these data indicated the capacity of PEV-DOX to transfer effectively DOX to cancer cells.

### Cytotoxic effects of fresh and cryopreserved DOX-loaded PLT on various cancer cell lines

**Fig. 7:**
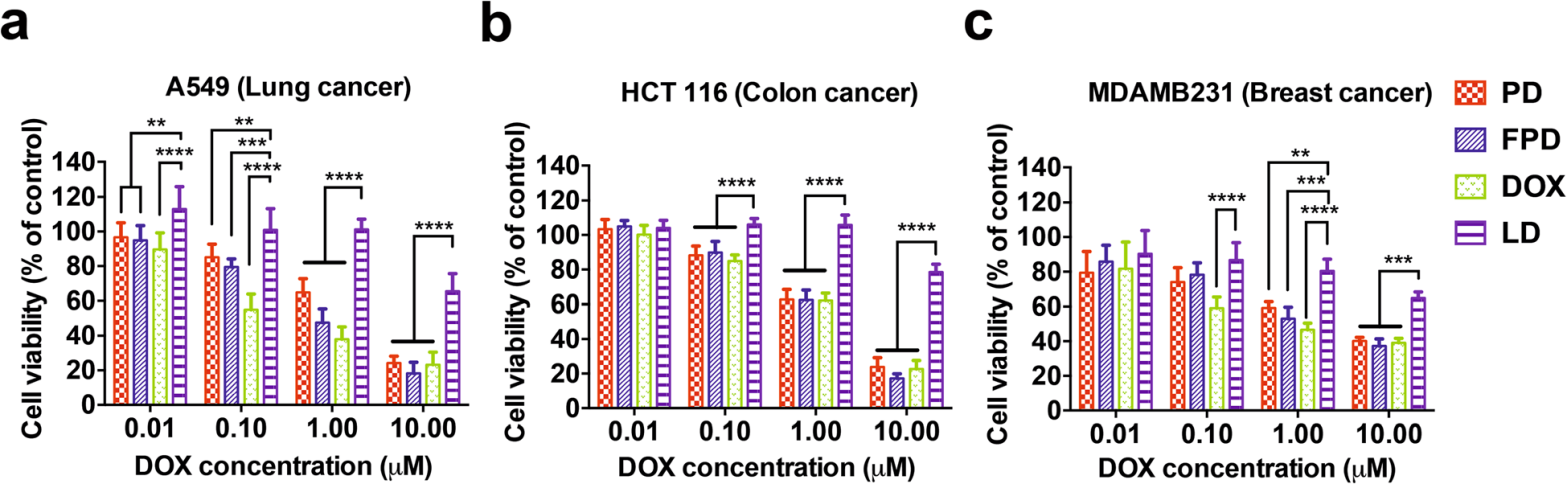
In vitro cytotoxicity of fresh and cryopreserved DOX-loaded PLT on cancer cell lines. (**a**) A549 lung cancer cells, (**b**) HCT 116 colon cancer cells, (**c**) MDA-MB-231 breast cancer cells were grown for 48 h in the presence of PD, FPD, DOX and LD. The viability was assessed with a CCK-8 assay. Cytotoxicity compared to liposomal DOX. ** *p* < 0.01, *** *p* < 0.001, **** *p* < 0.0001. *Abbreviations: PD: fresh DOX- loaded PLT, FPD: cryopreserved DOX-loaded PLT, DOX: doxorubicin, LD: liposomal doxorubicin*

To verify the scientific rationale of the “Trojan Horse” PLT carrier strategy against cancer cells, and evaluate any functional impairment associated with the storage freezing, we assessed the cytotoxicity of DOX-loaded PLT and cryopreserved DOX-loaded PLT. Viabilities of the A549 lung cancer cell line, HCT 116 colon cancer cell line and MDA-MB-231 breast cancer cell line exposed to DOX-loaded PLT, cryopreserved DOX-loaded PLT, free DOX (positive control), and liposomal DOX for 48 h were assessed by a CCK-8 assay. Cell medium, PLT and cryopreserved PLT used as negative controls were first confirmed to be essentially non-toxic at the doses used to three cancer cell lines. In contrast, DOX-loaded PLT and cryopreserved DOX-loaded PLT (10 μM) were significantly (*p* ≤ 0.0001) more toxic to both cancer cell lines than normal and cryopreserved PLT, confirming their capacity to release DOX. Cytotoxicities of DOX-loaded PLT and cryopreserved DOX-loaded PLT toward lung (Fig. 7a), colon (Fig. 7b), and breast cancer cells (Fig. 7c) were significantly less than that of DOX in the lowest range concentration, but similar at the highest concentration (10 μM). Liposomal DOX exerted a highly cytotoxic effect at the highest concentration (10 μM), but its effect remained limited and significantly (*p* < 0.0001) less than that of DOX-loaded PLT and cryopreserved DOX-loaded PLT; this finding suggests, as expected, a lack of stimulation of DOX release from liposomal DOX under these culture conditions due to a lack of responsiveness to biological triggering factors released by cancer cells. Mean IC50 values of free DOX were 0.23, 1.8, and 0.69 μM for A549, HCT 116, and MDA-MB-231 cells, respectively. Respective IC50 values of DOX-loaded PLT were 2.06, 1.94, and 2.37 μM of DOX-equivalents, for cryopreserved DOX-loaded PLT were 0.87, 1.7, and 1.52 μM, and for liposomal DOX were 20.09, 39.68, and 15.82 μM. While IC50 values for DOX-loaded PLT and cryopreserved DOX-loaded PLT were close to each other, and similar to that obtained with the equivalent amount of DOX, they were over 7~23-times lower than that achieved with liposomal DOX. Thus, these data indicated that DOX-loaded PLT and cryopreserved DOX-loaded PLT have stronger capacities to release DOX to the cancer cell environment in vitro than does liposomal DOX. The cytotoxicity of loaded PLT was much higher in breast, lung, and colon cell lines than that of liposomal DOX, evidencing the superior reactivity of loaded PLT to act as an environment-responsive “Trojan Horse” DDS. The cytotoxic efficacy of DOX-loaded PLT and cryopreserved DOX-loaded PLT was similar to that of free DOX, suggesting quick release of DOX from loaded PLT in contact with cancer cells, consistent with the deconvolution microscopic observations showing DOX transfer in vitro within 90 min (Fig. S2). We suspect, however, that DOX-loaded PLT efficacy is actually stronger than that of free DOX as release studies showed that only about 66.67% of DOX present in cryopreserved DOX-loaded PLT was released after 72 h of culture in the presence of cancer cells (Fig. 3d). This suggests that loaded PLT, due to their unique reactivity in contact to cancer cells, especially through the release of PEV, could exert stronger toxicity at a dose lower than that of free DOX.

## Discussion

Current therapeutic strategies of cancer rely upon combining surgery, chemotherapy, immuno and-chemotherapy, as well as radiation. Conventional chemotherapy using nucleotoxic agents is non-discriminative, and its use is associated with detrimental impacts on normal cells. Understandably, targeted DDS platforms could circumvent drawbacks of conventional therapies [46] by improving drug biodistributions, reaching tumour sites at effective concentrations, optimizing circulation half-lives, or decreasing side effects with improved efficacy [47, 48]. Liposomal DOX formulation does decrease the risks of drug resistance and helps improve drug solubility and stability, making drug formulation more straightforward [49]. Nevertheless, although cardiac toxicity is less pronounced, adverse events (skin reactions, peripheral neuropathy, and hypersensitivity reactions) are seen in 10%~ 15% of patients [49]. Polyethylene glycol (PEG), used to improve the blood residence time of such nanocarriers, is not immunologically inert: many patients have preexisting anti-PEG antibodies that may decrease the drug efficacy and tolerance [50, 51]. A critical drawback of synthetic nanoformulations is the lack of targeting, as drug delivery towards and diffusion within the tumour site depends only of the presumed enhanced permeability and retention effect [52]. Synthetic “undecorated” carriers are poorly equipped to overcome cellular barriers prior to releasing chemotherapeutic drugs [53], and only a mean 0.7% of drugs loaded into nanocarriers reach solid tumours [42]. Therefore, such a lack of targeting capacity explains the recent interest in using human cells as drug carriers, to take advantage of the inherent physiological targeting capacity provided by the physiological “decorations” of cell membranes by various glycoproteins [24, 30, 54, 55]. The potential to use, or mimic, circulatory cells as a DDS has been demonstrated in in vitro and in vivo models, providing a tool benefiting from the functional reactivity of cellular membranes, including PLT [23, 24, 56–58]. Circulatory cells are biocompatible and have long residence times in the blood circulation. Blood cells are sustainable cellular sources of DDS since, based on the most recent WHO survey, over 117.4 million blood collection procedures are performed each year in the world for transfusion medicine [59], providing a reliable supply. While most translational applications of blood cells have used red blood cell-based DDS so far [60], a focus on PLT has emerged recently [24, 61, 62]. PLT is a delicate cell to manipulate ex vivo as their smart lipid bilayer membrane embedded with glycoprotein integrins is highly reactive to other blood components, resulting in PLT activation, degranulation, and generation of PEV [63]. However, PLT being anucleated cells make them especially suitable for loading nucleotoxic anticancer cells agents; in addition, they expose membrane integrin receptors, such as GPIIb/IIIa, CD62P, and protease-activated receptors [17, 64] which interact with TF-releasing tumour cells [17] and thrombin, leading to TCIPA phenomenon [9, 17, 64] and PLT activation and degranulation. This PLT-cancer intimate connection is the very scientific rationale for using PLT as a cell-based targeted delivery platform of anticancer agents, following a “Trojan Horse” curative strategy [24, 61].

Our translational study is now the first to demonstrate that human PLT obtained by licensed collection procedures can be used to load DOX. We show that the resulting preparation can be stored frozen without altering the capacity to release DOX, which is essential for ease of clinical use. We documented a release mechanism of DOX based on an interaction between PLT and cancer cells involving EV and low pH. Over 50 clinical-grade human PC were collected following licensed procedures and found to meet specifications for transfusions [65]. The PC were centrifuged to isolate the PLT and remove plasma proteins as those are known to bind DOX [66] and could have affected the DOX loading procedure. DOX loading was achieved under mild incubations (1 h at 37 °C), as done by others [61, 62], but in PAS, as this solution is licensed for transfusion in many countries [67, 68]. PAS may decrease the risk of allergic transfusion reactions [69–71] in cancer patients eventually receiving a drug-loaded PLT-based therapy. We found that, under these conditions, the loading capacity of each PLT was as high as 15~36 × 10^6^ molecules of DOX and identified that loading was at its maximal 6 days after PC collection. This high loading is over 1000-fold more than in a 100-nm liposome (10,000~15,000 molecules/liposome) [72], thanks to PLT size and, probably, the unique capacity of entrapment of DOX through the canalicular system [73–75]. For ease of clinical treatment, it is important that the PLT formulation can undergo long-term storage. Loaded PLT could be stored frozen at − 80 °C and thawed in the presence of 6% DMSO cryoprotectant with a DOX yield of over 80%. We selected DMSO as it is already approved to freeze PLT for transfusion for emergency application by the military [34, 35] and for supportive treatment of patients with leukemia [76]. Recent clinical studies report the safety of DMSO-cryopreserved platelet concentrates for transfusion [77]. The mild manufacturing process preserved the morphology and functionality of the DOX-loaded PLT, as evidenced by (a) SEM observations, (b) maintained expression of important PLT markers by Western blotting, and (c) thromboelastography assay. In addition, the release kinetics of DOX from PLT in vitro was similar to that observed with artificial nanocarriers [78–81], and was significantly faster in acidic pH conditions mimicking the tumour microenvironment. The reasons for accelerated release of DOX by PLT at pH 5.5 is unclear and may involve several mechanisms. Acidic conditions have been shown to affect part of PLT reactivity, for instance a stronger expression of P-selectin [82] (a protein normally present on the membrane of the alpha-granules) that may also trigger DOX release. Low pH is associated with a change in PLT structure [83] that may affect the “permeability” of the open canalicular system [84] and increase the discharge of DOX. Finally, a low pH typical of the tumour microenvironment can trigger the disruption of PEV-DOX, as it has been shown to occur with cancer cell-derived vesicles [85]. Interestingly, cancer cell-derived TF-EVs significantly accelerated the release of DOX by inducing PLT activation and degranulation. This is consistent with the existence of a cancer cells/PLT mutual activation loop triggered by TF released by cancer cells [11, 86]. The enhanced release of DOX from PLT by both low pH and TF-EV reveals a dual release mechanism of DOX from PLT carriers in conditions mimicking the tumour environment. In addition, the loaded PLT formulation was capable of reacting to thrombin-induced activation and generating PEV. Such PEV, with a size close to 200 nm, could act as an instrumental secondary DDS of DOX to cancer cells with a stronger capacity to infiltrate the tumour microenvironment and fuse with cancer cells [45].

The cytotoxicity of loaded PLT against cancer cell lines was much higher than that of Lipo-DOX, evidencing their superior ability to act as a “Trojan Horse” DDS. DOX-loaded PLT efficacy was similar to that of free DOX, suggesting quick release of DOX from loaded PLT in contact with tumour cells, consistent with the deconvolution microscopic observations showing DOX transfer in vitro within 90 min (Fig. S1). Others found superior efficacy of freshly prepared DOX-loaded PLT than free DOX in both in vitro and in vivo mice cancer models [61]. However, our data are globally consistent with the release kinetic study that showed that about 66% of the initial DOX content was released after 72 h. This suggests that this loaded PLT formulation could exert a sustained toxicity, and at a dose lower than that of free DOX.

## Conclusions

**Fig. 8:**
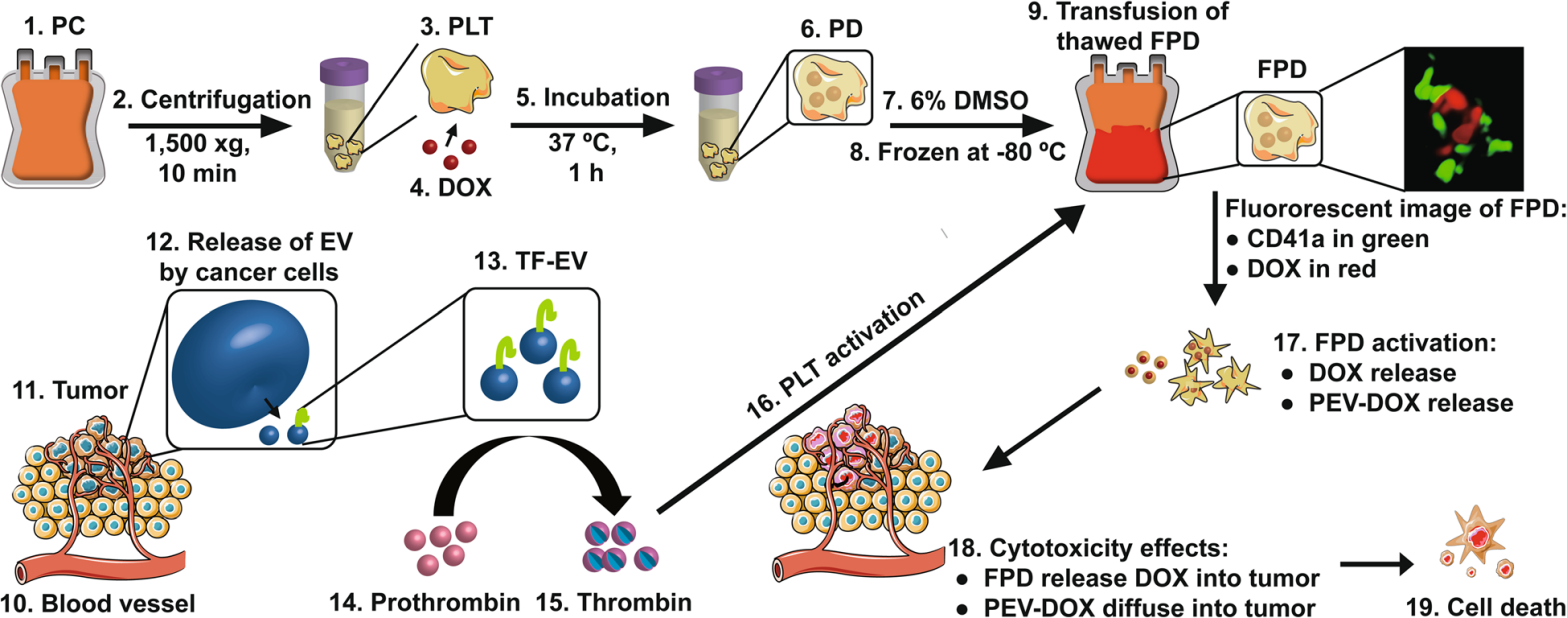
Therapeutic concept and mode of action of the “Trojan horse” treatment using PLT and PEV as a drug delivery system. PC is collected from patients or donors then centrifuged at 1500 xg for 10 min to pelletize PLT. PLT is incubated with a DOX solution in PAS for 1 h to generate DOX-loaded PLT. DOX-loaded PLT is frozen in the presence of 6% DMSO following approved procedures and stored at − 80 °C until use. After thawing, a therapeutic dose of cryopreserved DOX-loaded PLT is transfused to the patient. Through the blood circulation, the cryopreserved DOX-loaded PLT reaches the tumour micro-environment where cancer cells release pro-coagulant factors and EV involving TF-EV. TF-EV convert prothrombin in blood to thrombin resulting in PLT activation. Cryopreserved DOX-loaded PLT activation in the tumour microenvironment contributes to the release of DOX and of PEV-DOX that induce cancer cell death. The figure of tumour with blood vessel was prepared using Servier Medical Art (www.servier.com). *Abbreviations: PLT: platelet, PEV: PLT-derived extracellular vesicle, PC: PLT concentrate, PAS: PLT additive solution, DMSO: dimethyl sulfoxide, TF-EV: EV expressing tissue factor*

Our study supports the strong translational feasibility of using collected PLT as a smart cellular carrier of anti-cancer drug, and contributes to understanding the drug releasing mechanisms involved in the tumour microenvironment. Thus, as summarized and conceptualized in Fig. 8, preserved PLT membrane receptors (such as GPIIb/IIIa) serve as a targeting arm to reach tumour sites through the bloodstream, as already demonstrated in in vivo models. In the tumour microenvironment, the acidic pH, the presence of cancer cells and TF-EV, and the thrombin-rich milieu, which is typical of all tumours, contribute to PLT activation, PEV generation, and release of DOX, in a complementary cancer cell killing loop. Our study demonstrates the role that PLT and PEV play by being a potent delivery system of DOX, and presumable other hydrophilic anti-cancer agents, in a universal “Trojan Horse” suicide strategy stimulated by cancer cells themselves. Our work further demonstrates the scientific and medical rationale of this strategy as we used clinical grade PC as starting materials, and solutions like PAS and DMSO that are already licensed for transfusion purposes to patients.

From our’s and others’ data [61, 62], clinical translation can be envisaged using as source material PLT collected from either cancer patients themselves or allogeneic healthy donors. PLT could be obtained using the currently licensed apheresis machines and collection procedures in a hospital setting and using PAS as PLT storage solution. DOX loading would require the development of a dedicated single-use medical device allowing aseptic addition, efficient encapsulation and subsequent washing to eliminate the free DOX, before sterile-docking addition of DMSO and freezing. Additionally, our data suggest the possibility to use DOX-loaded PEV as DDS, following the current trends seen in therapeutic applications of EVs [87]. DOX-loaded PEV is a current focus of our research work that includes defining optimal loading conditions and formulation, and stability studies evaluating release profile as well as lack of aggregation and morphological changes.

## Supplementary information

**Additional file 1: Table S1.** Effects of storage conditions of PC on the loading capacity of DOX into PLT.

**Additional file 2: Fig. S1.** Time-lapse video using deconvolution microscopy demonstrates the capacity of DOX-loaded PLT to transfer DOX to MCF-7 breast cancer-derived cells.

**Additional file 3: Fig. S2.** Cell proliferation assay of breast cancer cells treated with fresh DOX-loaded PLT compared to other drugs.

**Additional file 4: Fig. S3.** Morphology of cryopreserved DOX-loaded PLT.

**Additional file 5: Fig. S4.** The stability test of DOX-loaded PLT stored in PAS for up to 6 days.

**Additional file 6: Fig.S5.** MP-TF activity of conditioned medium cultured with MDA-MB-231 breast cancer cells.

**Additional file 7: Fig. S6.** Size distributions and images of PEV and PEV-DOX.

**Additional file 8: Table S2.** Western blot antibody information.

## Data Availability

All materials are available from the corresponding author.

## Abbreviations

BSA: Bovine serum albumin
CD41: PLT Glycoprotein IIb
CD61: PLT Glycoprotein IIIa
CD62P: P-selectin
CM: Conditioned medium
DDS: Drug delivery system
DMSO: Dimethyl sulfoxide
DOX: Doxorubicin
GAPDH: Glyceraldehyde 3-phosphate dehydrogenase
HPLC: High performance liquid chromatography
NTA: Nanoparticle tracking analysis
PAR-1: Protease activated receptor-1
PAS: Platelet additive solution
PBS: Phosphate-buffered saline
PC: Platelet concentrate
PEV: PLT-derived extracellular vesicle
PLT: Platelet
SDS: Sodium dodecyl sulfate
SEM: Scanning electron microscopy
TCIPA: Tumour cell-induced PLT aggregation
TEG: Thromboelastography
TF-EV: Extracellular vesicle expressing tissue factor
