# Supplementary figures and images for: Clinical-grade cryopreserved doxorubicin-loaded platelets: role of cancer cells and platelet extracellular vesicles activation loop
Source: J Biomed Sci. 2020 Mar 23;27:45. doi: 10.1186/s12929-020-00633-2 (PMC7087392; doi:10.1186/s12929-020-00633-2)

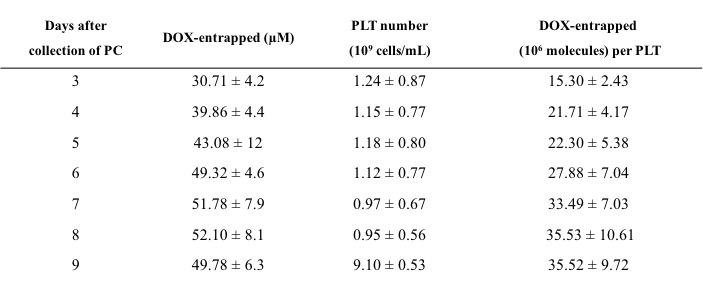

Supplement: Supplementary file 1 — Additional file 1: Table S1. Effects of storage conditions of PC on the loading capacity of DOX into PLT. [file 12929_2020_633_MOESM1_ESM.jpg]

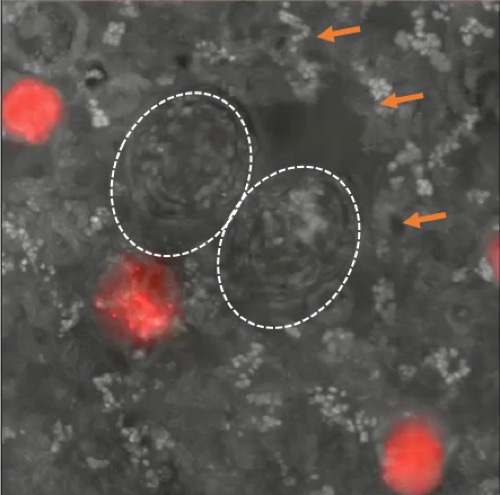

Supplement: Supplementary file 2 — Additional file 2: Fig. S1. Time-lapse video using deconvolution microscopy demonstrates the capacity of DOX-loaded PLT to transfer DOX to MCF-7 breast cancer-derived cells. [file 12929_2020_633_MOESM2_ESM.zip › Figure S1. Cellular uptake of DOX-loaded PLT.jpg]

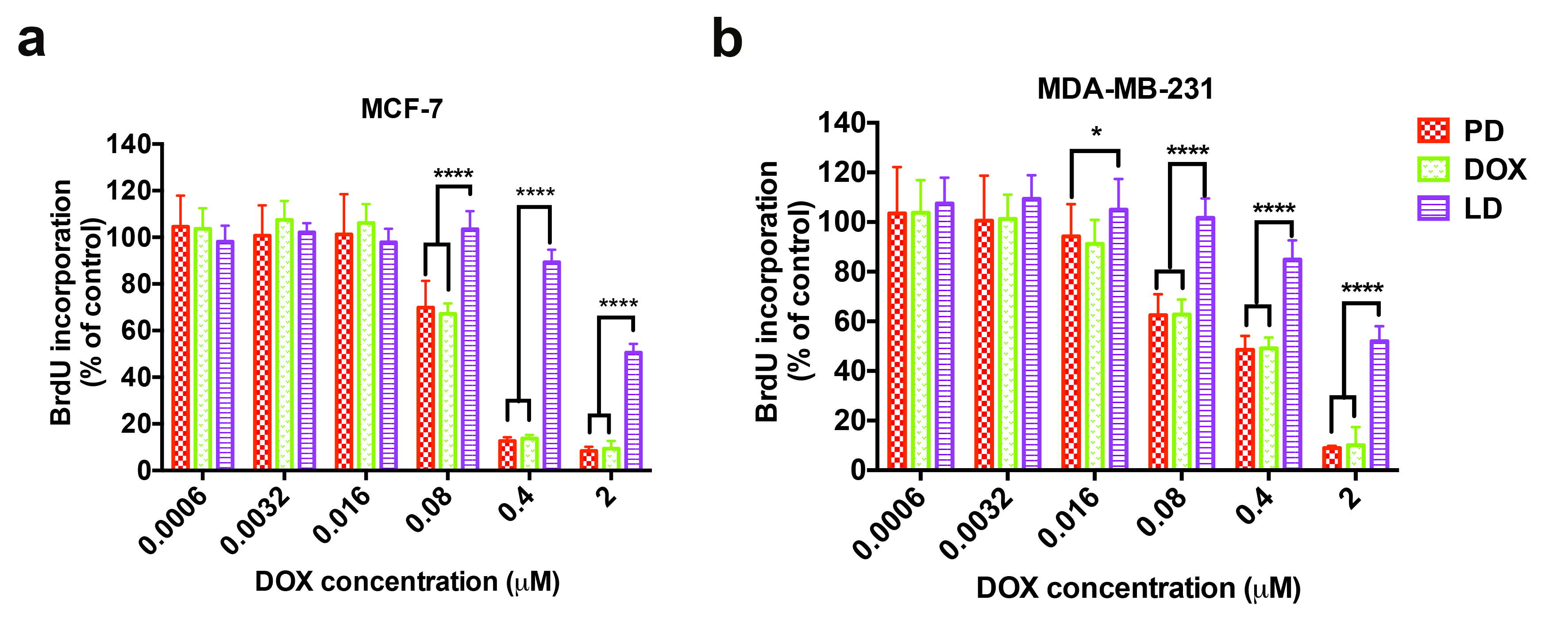

Supplement: Supplementary file 3 — Additional file 3: Fig. S2. Cell proliferation assay of breast cancer cells treated with fresh DOX-loaded PLT compared to other drugs. [file 12929_2020_633_MOESM3_ESM.tif]

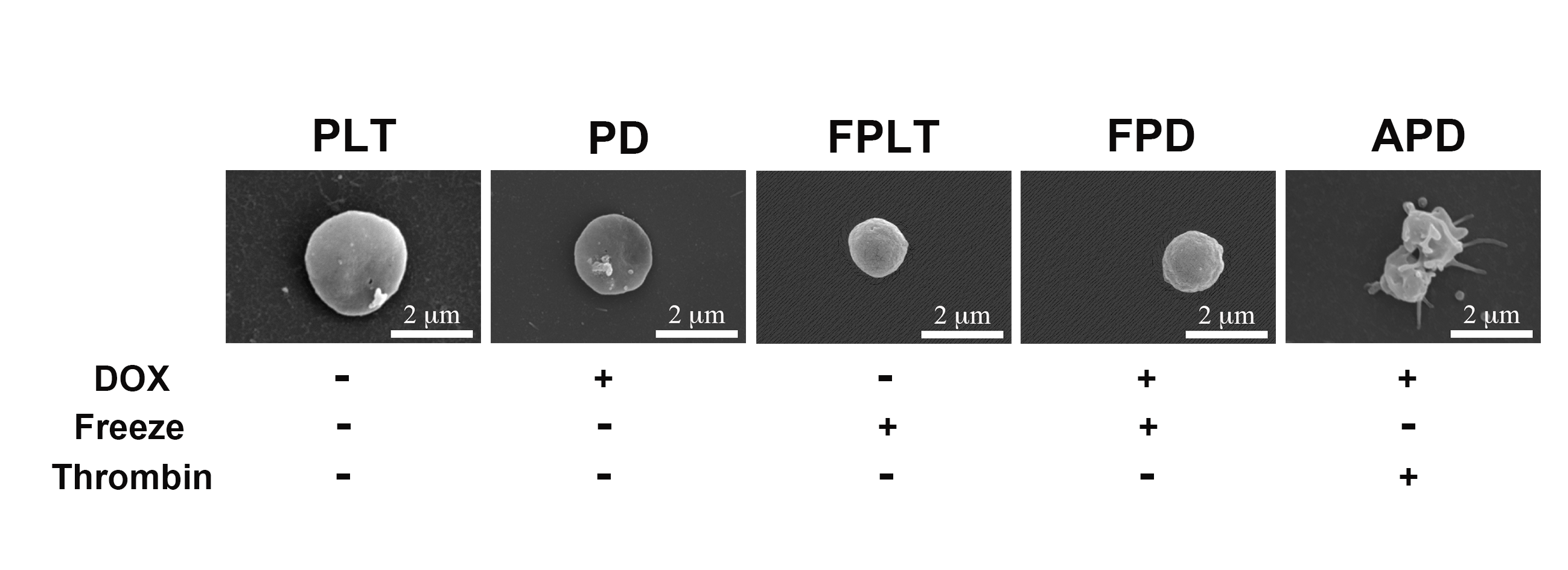

Supplement: Supplementary file 4 — Additional file 4: Fig. S3. Morphology of cryopreserved DOX-loaded PLT. [file 12929_2020_633_MOESM4_ESM.tif]

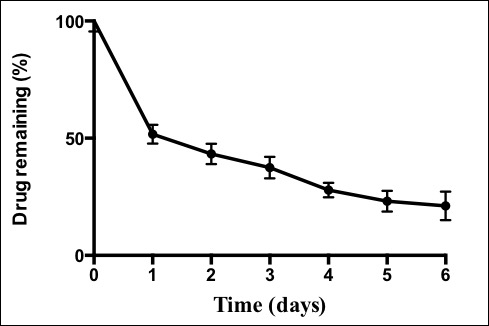

Supplement: Supplementary file 5 — Additional file 5: Fig. S4. The stability test of DOX-loaded PLT stored in PAS for up to 6 days. [file 12929_2020_633_MOESM5_ESM.jpg]

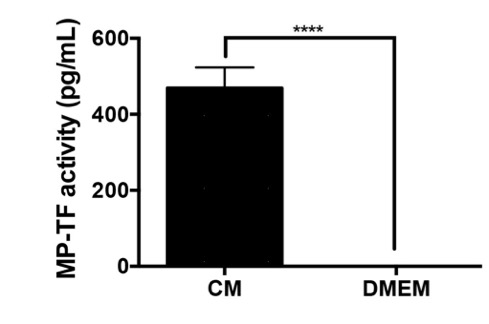

Supplement: Supplementary file 6 — Additional file 6: Fig.S5. MP-TF activity of conditioned medium cultured with MDA-MB-231 breast cancer cells. [file 12929_2020_633_MOESM6_ESM.jpg]

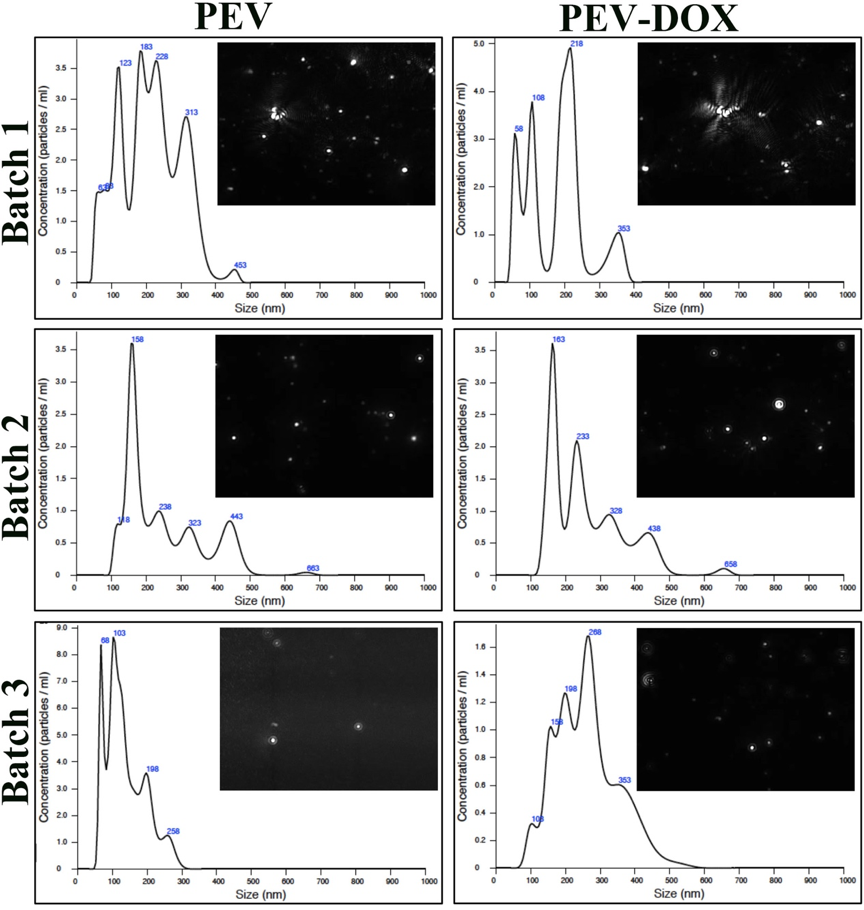

Supplement: Supplementary file 7 — Additional file 7: Fig. S6. Size distributions and images of PEV and PEV-DOX. [file 12929_2020_633_MOESM7_ESM.png]

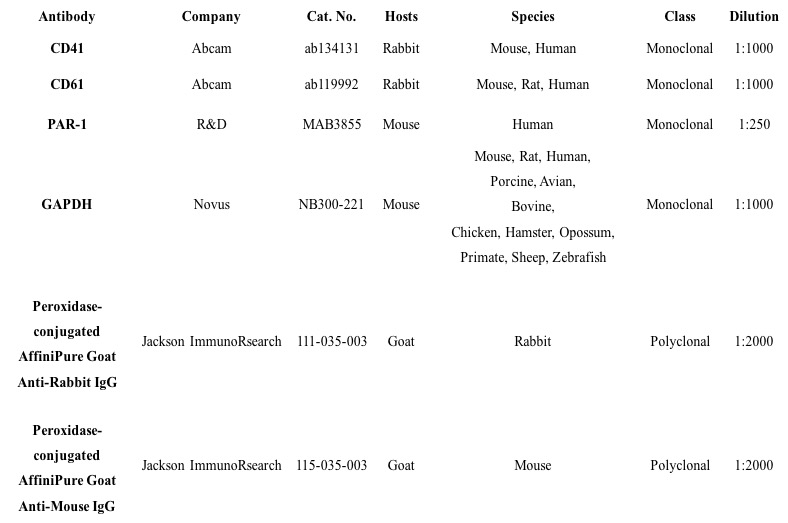

Supplement: Supplementary file 8 — Additional file 8: Table S2. Western blot antibody information. [file 12929_2020_633_MOESM8_ESM.jpg]
